# The interplay between bone healing and remodeling around dental implants

**DOI:** 10.1038/s41598-020-60735-7

**Published:** 2020-03-09

**Authors:** Soroush Irandoust, Sinan Müftü

**Affiliations:** Department of Mechanical and Industrial Engineering, Northeastern University, Boston, MA 02115 USA

**Keywords:** Biomedical engineering, Implants

## Abstract

Long-term bone healing/adaptation after a dental implant treatment starts with diffusion of mesenchymal stem cells to the wounded region and their subsequent differentiation. The healing phase is followed by the bone-remodeling phase. In this work, a mechano-regulatory cellular differentiation model was used to simulate tissue healing around an immediately loaded dental implant. All tissue types were modeled as poroelastic in the healing phase. Material properties of the healing region were updated after each loading cycle for 30 cycles (days). The tissue distribution in the healed state was then used as the initial condition for the remodeling phase during which regions healed into bone adapt their apparent density with respect to a homeostatic remodeling stimulus. The short- (bone healing) and long-term (bone remodeling) effects of initial implant micromotion during the healing phase were studied. Development of soft tissue was observed both in the coronal region due to high fluid velocity, and on the vertical sides of the healing-gap due to high shear stress. In cases with small implant micromotion, tissue between the implant threads differentiated into bone during the healing phase but resorbed during remodeling. In cases with large implant micromotion, higher percentage of the healing region differentiated into soft tissue resulting in smaller volume of bone tissue available for remodeling. However, the remaining bone region developed higher density bone tissue. It was concluded that an optimal range of initial implant micromotion could be designed for a specific patient in order to achieve the desired long-term functional properties.

## Introduction

Dental implant fixtures have become an integral part of treatment for partially or fully edentulous patients^1^ since Branemark introduced the two-stage treatment protocol^2^. More recently, a single-stage protocol, where the implant is surgically inserted, the prosthetic tooth installed and the implant immediately loaded, is considered beneficial as it reduces the number of surgical interventions. Osseointegration of immediately loaded implants has been the subject of numerous clinical and animal studies. Provided that the primary stability of the implant can be ensured^3,4^, immediate loading has been shown to be a reliable treatment^1,5^, without disturbing the biological osseointegration process^6^ or affecting bone mineral apposition rate^7^. In general, the primary stability is ensured by splinting the new prosthetic tooth to a stable anchor point and/or by making sure that the implant threads engage with existing bone. Even under these conditions, high occlusal loading is considered as a risk factor for immediately loaded implants^5^.

Small holes drilled in bone heal by intramembranous ossification in the first few weeks after surgery^8^. Such bone formation starts with blood clot formation, vascularization within the healing gap, and proliferation and migration of mesenchymal stem cells (MSCs) from surrounding bone marrow^9^. Under favorable conditions and stable sites, MSCs differentiate into osteoblasts and woven bone forms through osteogenesis^10^ followed by compaction of woven bone. After about a month^9,11^, bone remodeling starts. In this phase, bone continuously adapts itself by adjusting its apparent mass density to mechanical loading and functionality^12,13^. Similarly, dental implant surgery involves placement of an implant in a drilled hole (osteotomy). The healing mechanism of the tissue in the gap between the implant and the preexisting bone was shown to be intramembranous bone formation^10,14^.

As stated above, in clinical treatments implants are assured to have initial contact with the surrounding bone to avoid excessive micromotion^15^. While the primary stability of the implant helps with the success of the treatment, it is still not clear how much micromotion can be tolerated without causing fibrous tissue encapsulation^16^ especially because each treatment scenario is patient specific. In order to address this question experimental studies were conducted, where the implant was inserted in an osteotomy wider than itself without any initial bone anchorage, while the implant micromotion was controlled during the experiment^14,17^. Such studies shed light on the interaction of micromotion and bone healing at a fundamental level. In this work, a mathematical model of a similar insertion site, where there is no initial bone-to-implant contact is created in order to understand the direct effects of implant micromotion on bone healing.

The independent variable to measure the effects of external loading on the healing tissue has historically been displacement, because it is easier to control micron-level displacements in experiments performed on soft tissue. In fact, studies of healing around immediately loaded implants typically use implant micromotion to assess healing pathway^17^. On the other hand, the independent variable to measure bone response and remodeling has historically been force, because bone can handle relatively large forces^18^. In the present work, the same practice is followed in order to be consistent with the experimental literature; during the healing phase, micromotion of the implant is used as the independent variable, whereas during the remodeling phase mastication force is used. It is of course interesting to note that in a micromotion-controlled environment the tissue properties change continuously and the load carrying capacity of the tissue adjusts accordingly.

Clinical and experimental examinations create the opportunity to observe biological processes of fracture healing^9,19,20^ and bone remodeling^21^; and, in-silico studies can represent how biological factors contribute to the outcome of a dental implant treatment^22^. Numerous computer simulations have been carried out to investigate effects of mechanical loading on bone healing and bone remodeling^18,23–32^. In healing studies, long-term adaptation of the bone tissue is not investigated, and remodeling studies usually do not start from a realistic initial state. To the best of our knowledge, this is the first study modeling both biological processes consecutively.

## Results

**Figure 1:**
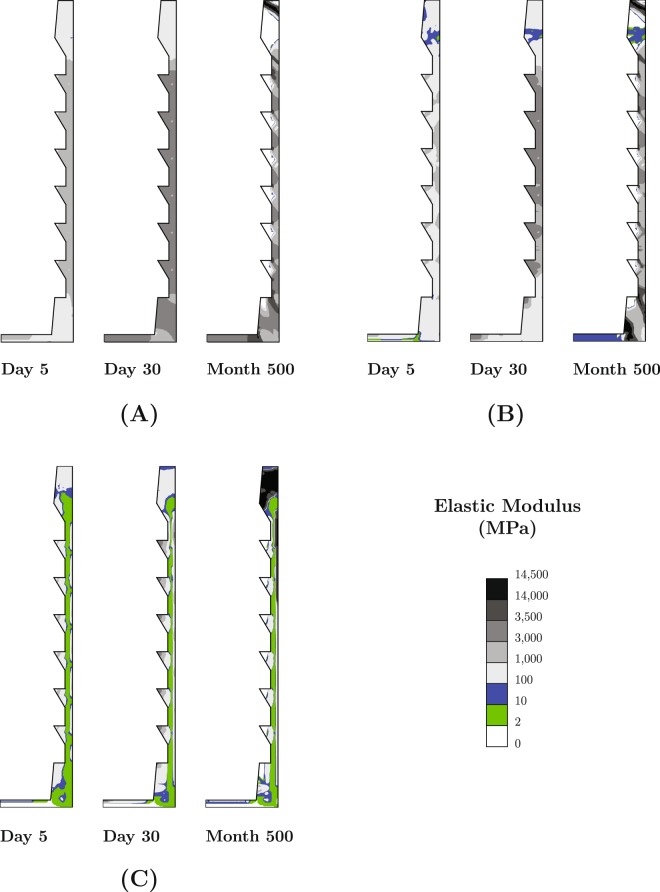
Transient change of local elastic modulus in the healing region, for **(A)** z_max_ = 5 μm, **(B)** z_max_ = 10 μm, and **(C)** z_max_ = 20 μm.

Transient change of elastic modulus during healing and remodeling for different ranges of initial implant micromotion (during the healing phase) is shown in Fig. 1. It is interesting to note that regardless of the micromotion range, the tissue between the implant threads develop into bone during the healing phase (days 1–30) but resorb during remodeling. The fate of the tissue on the vertical sides of the healing gap strongly depends on the micromotion amplitude.

**Figure 2:**
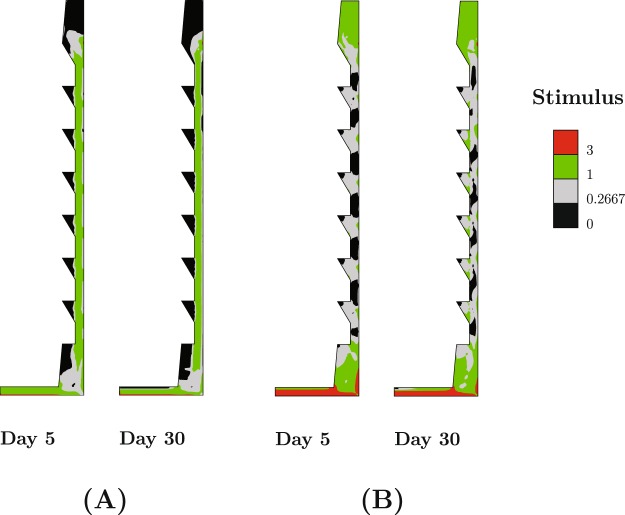
Distribution of (**A**) solid stimulus and (**B**) fluid stimulus in the healing region at day 5 and day 30 (healing phase) for z_max_ = 20 μm.

The distributions of the solid and fluid stimuli in the healing gap are shown in Fig. 2 for days-5 and −30. This reveals that the regions in between implant threads experience the lowest solid stimulus (lowest shear strain) and lowest fluid velocity compared to the other regions of the healing gap. The regions between the implant threads have smaller shares of transferring mechanical load to surrounding cancellous bone. This characteristic leads these regions to a faster healing during the healing phase, but to resorption later during remodeling phase. Resorption due to insufficient remodeling stimulus is known as stress shielding^33^. During the healing phase, large loading amplitude (z_max_ = 20 μm) causes soft tissue development on the vertical sides of the healing gap and in the coronal region (Fig. 1). This observation is correlated with the high solid and fluid stimuli in these regions (Fig. 2). Note that the high fluid velocity in the coronal region is due to the very low permeability of the adjacent cortical bone.

**Figure 3:**
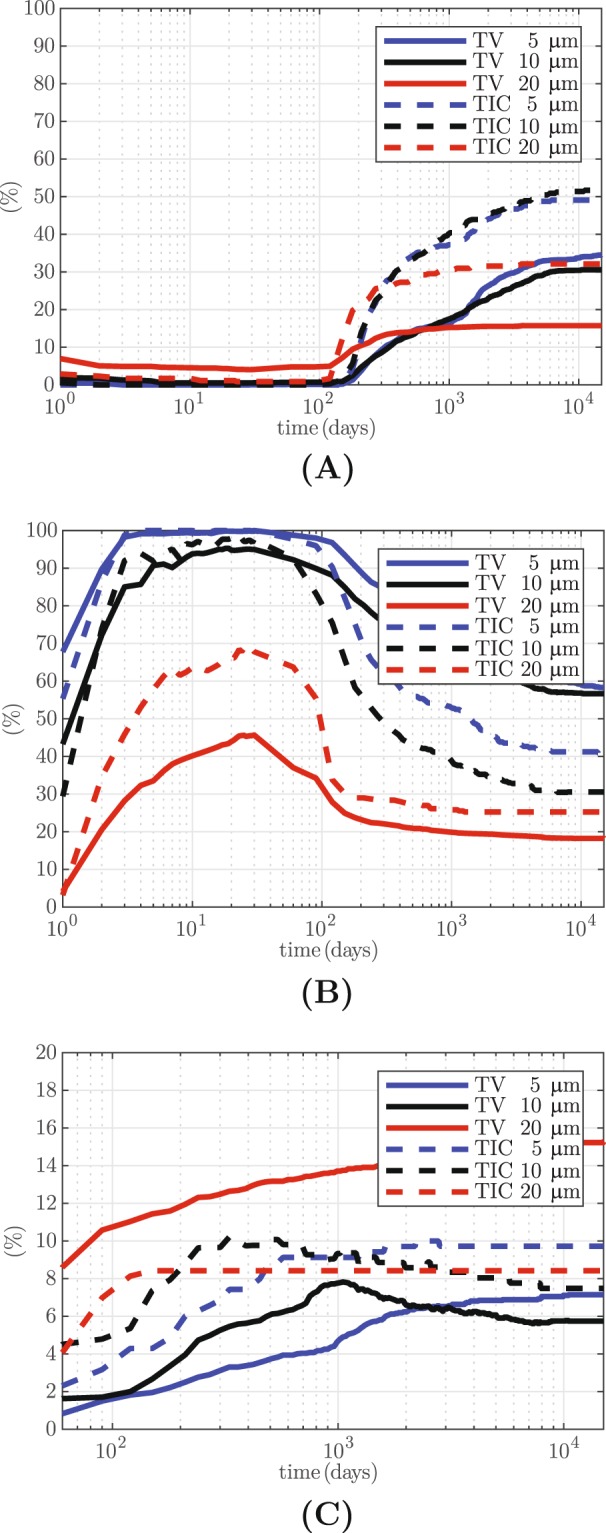
Variation of the proportions of **(A)** fibrous tissue (E <2 MPa), **(B)** woven and trabecular bone (100 MPa <E <3.5 GPa) and **(C)** cortical bone (E >3.5 GPa) during healing (t <30 days) and remodeling (t >30 days) as measured by TV and TIC. Results are given for z_max_ values of 5, 10 and 20 μm.

Two metrics are defined in order investigate tissue evolution during healing and remodeling phases. Tissue volume (TV) is the ratio of the volume of a specific tissue type to the volume of the healing region. Tissue-to-implant contact (TIC) shows how much of the implant surface is in contact with a specific tissue type. TV and TIC histories for soft tissue (E <2 MPa), immature and trabecular bone (100 MPa <E <3.5 GPa) and cortical bone (E >3.5 GPa) are presented in Fig. 3 for a total of 15,000 days after start of treatment. Note that healing is simulated in the first 30 days (t <30), after which the simulation algorithm is switched to remodeling (t >30).

These results reveal interesting interplay between the different tissue types in the longitudinal simulations. The case subjected to 5 μm motion is predicted to develop negligible fibrous tissue (Fig. 3A, TV = 0%, TIC = 0%) and nearly 100% immature and trabecular bone (Fig. 3B) at the end of the healing period. During the remodeling phase, some of the woven and trabecular bone (Fig. 3B) resorb into soft tissue (Fig. 3A, TV = 35%, TIC = 50%) and some develop into cortical bone (Fig. 3C, TV = 7%, TIC = 10%). During this phase, resorption is attributed to stress shielding effect, while development of the cortical bone is attributed to the remodeling process seeking a higher bone density, in order to respond to the 100 N of mastication load. Similar predictions are made for the implant that was subjected to 10 μm motion during the healing phase. This treatment is also predicted to result in negligible fibrous tissue (Fig. 3A), slightly lower volume (95%) of immature and trabecular bone (Fig. 3B) at the end of the healing phase, and a similar trend and amount of bone resorption (Fig. 3A) and densification (Fig. 3C) in the long term (t >30).

Tissue around the implant that is subjected to 20 μm motion evolves differently, resulting in a weaker prognosis for the simulated treatment. This implant develops a relatively large amount of fibrous tissue (Fig. 3A, TV = 5%, TIC = 0.9%) and cartilaginous tissue (TV = 45.8%, TIC = 27.3%) and a relatively low amount of woven and trabecular bone (Fig. 3B, TV = 45%, TIC = 68%) at the end of the healing period. During the remodeling phase some of the woven and trabecular bone develops into cortical bone (Fig. 3C, TV = 15%, TIC = 8%). Some low stiffness fibrous tissue is also developed due to stress shielding (Fig. 3A, TV = 16%, TIC = 32%). A steady state for the cortical bone is not quite established at the end of the simulated remodeling phase.

The TV and TIC values at the end of the healing (t = 30 days) and remodeling (t = 15,000 days) periods are summarized in Table 1. A clear correlation between magnitude of the implant motion in the first 30 days and the amount of bone that is maintained very much longer after the initial healing period is predicted. This shows that after 15,000 days 65%, 63% and 33% of the TV consists of either trabecular or cortical bone, for implant motion values of 5, 10 and 20 μm, respectively. For the lower two values of the micro-motion, the tissue is primarily trabecular bone, whereas for the 20 μm motion, the distribution is 18% trabecular and 15% cortical bone. Thus it is seen that, in this case, the tissue is able to recover load carrying capacity by redistributing the bone quality from 46% woven and trabecular bone at the end of the healing period to 18% trabecular and 15% cortical bone.

**Table 1:** Predicted values of TV and TIC at the end of the healing and remodeling periods.

	Fibrous tissue E < 2 MPa		Woven & trabecular bone 0.1 < E < 3.5 GPa		Cortical bone E > 3.5 GPa	
	TV (%)	TIC (%)	TV (%)	TIC (%)	TV (%)	TIC (%)
**t = 30 days**						
5 μm	0.0	0.0	100	100	0.0	0.0
10 μm	0.5	0.0	95	97.7	0.0	0.0
20 μm	4.1	0.9	45.7	67.8	0.0	0.0
**t = 15 × 10**^**3**^ **days**						
5 μm	34.5	49.6	58.3	40.7	7.1	9.7
10 μm	30.6	51.7	56.7	30.6	5.7	7.5
20 μm	15.7	32.1	18.2	25.3	15.3	8.4

## Discussion

In general, mechanical loading is in favor of formation of high-density bone during remodeling, but it is in favor of development of soft tissue during bone healing. The bone healing and remodeling theories, both of which are rooted in empirical observation, lead to this outcome. This work demonstrates the interplay between healing, remodeling and loading levels and shows that the point in time where bone quality is measured has a major role in the evaluation of the peri-implant osseointegration. This observation perhaps sheds light onto the seemingly contradictory results obtained in clinical and experimental studies involving animals.

There are numerous such studies which show that the long-term success of implant treatments depends on the mechanical conditions during the healing phase. Sagara *et al*. observe low levels of direct bone contact in histological examination and attribute this to early loading and excessive micromotion^34^. On the other hand, Piatelli *et al*. indicate immediate loading to be in favor of more bone implant contact (BIC)^35^ and Henry *et al*. attribute more mature cortical bone around the implant to early loading^36^ (Fig. 3C shows the same behavior). Presence and quality of bone surrounding an implant^37^ and its initial stability^3,4^ have also been extensively mentioned as important determinants of outcome of dental implant treatments. Excessive loading and relative motion of the implant are mentioned as important factors in development of interfacial fibrous tissue^37–39^, which can be seen in Fig. 1C as well. To the contrary, Duyck *et al*. found that low micromotion is less favorable than a high micromotion^19^. This appears to be in agreement with evolution of TV in Fig. 3A.

Primary stability of the implant depends also on the insertion torque (IT) and the extent of initial BIC. High IT is expected to result in less implant micromotion. Cha *et al*.^21^ showed that implants with high IT cause a wider dead zone of osteocytes at the implant interface. On the other hand, Grandi *et al*. showed that using high IT does not prevent osseointegration^20^. The effect of the dead zone and simulation of the IT are beyond the scope of this work but should be included in future studies.

It is important to realize that for a given mastication force on an implant, there will be varying ranges of implant micromotion from patient to patient depending on the quality of the surrounding bone and the initial bone implant contact. While the current model does not start from a realistic implant insertion scenario in clinical treatment sense (no initial BIC), it sheds light onto the intricate dependence of the long-term bone maintenance on the earlier healing phase. Moreover, the micromotion threshold can be translated into tissue level variables. This work showed that for largest micromotion value during healing (z_max_ = 20 μm), octahedral shear strain values of %3.75 and higher cause soft tissue formation along the vertical sides of the healing gap; whereas in the coronal region of the gap and under the implant, combination of high shear strain (%3.75 and higher) and high fluid velocity (3 μm/s and higher) are the reasons for soft tissue development. The fastest fluid velocity is observed under the implant with values exceeding 9 μm/s.

Simulation of the entire treatment duration by using the existing bone healing and remodeling models in a sequential manner explains the apparent inconsistencies reported in clinical studies; and helps demonstrate how interrelated and complex this treatment modality can be. In particular, it is seen that reaching a bone mass distribution that appears favorable at the end of a three- or four-week long healing period may not be an indicator of the long-term bone maintenance. The entire healing and remodeling process should be considered to this end. On the other hand, as expected, this work confirms that a healing period that results in low quality/quantity is not indicative of long-term failure of the treatment.

## Summary and Conclusion

Simulations of bone healing followed by bone remodeling were carried out in order to contribute to our understanding of long-term osseointegration and bone remodeling around dental implant systems. While the biology of wound healing and remodeling around oral implants is a complex phenomenon and it is still under investigation, in this study these processes are assumed to be the same as in long bone. The work shows that evolution of tissue type following an implant treatment does not have a linear correlation with mechanical usage (i.e. micromotion levels). Moreover, the end state of bone healing which transitions to remodeling plays a crucial role in the distribution of different tissue types around the implant in the long term. Without considering the healing process, higher mechanical usage would guide the predictions toward a higher bone density and cannot predict development of soft tissue in the presence of excessive mechanical loading. On the other hand, studying only the healing phase does not provide any information about the long-term adaptation of internal apparent bone density and potential regions of bone resorption. This work shows that an optimal range for implant micromotion and for a given implant contour should be possible, particularly on a patient specific basis, in order to achieve the desired outcome and functionality for dental implant treatments.

## Materials and Methods

**Figure 4:**
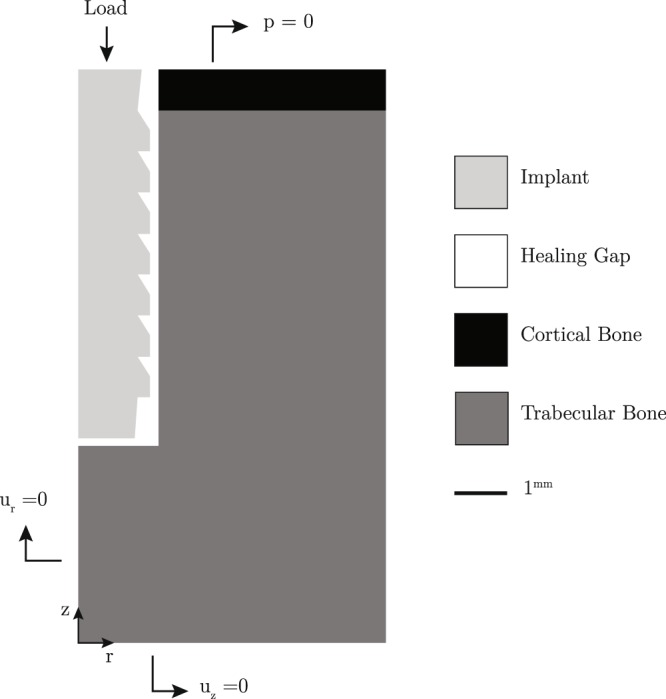
Cross-section depicting a dental implant with buttress type threads surrounded by cortical and cancellous bone types and the healing gap.

In order to study peri-implant bone healing and the subsequent bone remodeling, an axisymmetric model (Fig. 4) of the bone and the implant was developed. The model includes a dental implant with inner and outer radius of 1.75 and 1.95 mm and height of 9 mm, cortical and cancellous bone regions, and the healing gap.The healing gap is 200 μm wide in which tissue properties evolve during healing and remodeling processes. In the healing phase, the bone and the tissue in the gap were modeled as poroelastic materials with the properties given in Table 2. Physics of a saturated porous medium such as the one present in the healing gap is governed by fluid mass conservation as well as equations of elastic equilibrium^40^. In addition to the two material constants (elastic modulus and Poisson’s ratio) defining the elastic behavior of the solid part of the healing region, four other material constants (dynamic permeability of the fluid, porosity, and solid and fluid bulk moduli) are required for the fluid mass conservation. The boundary conditions were defined to represent ambient pore pressure (p = 0) at the superior aspect of the cortical bone and the gap, and axisymmetry along the central axis of the system (Fig. 4). Cyclical, displacement-controlled loading was applied to the top of the implant along the implant (-z) axis. During the remodeling phase, all tissue types were assumed to behave in linear-elastic manner, where the physical deformation is governed solely by the equations of elastic equilibrium. The boundary conditions were kept the same as before. An axially oriented mastication load of 100 N was applied in the -z direction.

### Bone healing

Bone healing is a physiologically complex process, which can go through various healing pathways based on mechanical and biological factors^41^. The initial response to fracture in bone starts with migration of MSCs to the healing gap^42^. Lacroix and Prendergast suggested a random movement of stem cells (SCs) from a vascularized origin with maximum cell concentration toward the healing region^23^, governed by the diffusion equation:

1 $${\rm{D}}{\nabla }^{2}{{\rm{n}}}_{{\rm{sc}}}=\frac{{{\rm{dn}}}_{{\rm{sc}}}}{{\rm{dt}}}$$

where D is the diffusion constant, n_sc_ is local percentage of available stem cells, and t is the time. Note that at the beginning of fracture there are no stem cells in the healing region (i.e. ${{\rm{n}}}_{{\rm{s}}{\rm{c}}}=0\,\text{at}\,{\rm{t}}=0)$. D is calibrated such that n_sc_ reaches its maximum (${{\rm{n}}}_{{\rm{sc}}}^{({\rm{\max }})}=100$) in the entire healing region after 14 days^43–45^. In this work ${\rm{D}}=0.023\,\frac{{{\rm{mm}}}^{2}}{{\rm{day}}}$ was used.

Two biophysical stimuli, octahedral shear strain (γ) and interstitial fluid velocity (v) are thought to regulate cellular differentiation pathway^46,47^. The healing stimulus S is formulated as:

2 $${\rm{S}}=\frac{{\rm{\gamma }}}{{\rm{a}}}+\frac{{\rm{v}}}{{\rm{b}}}$$

where a = 0.0375 and b = 3 μm/s are two constants determined empirically^23^. Based on this regulatory model, higher values of S are described as the reason for fibrous tissue generation, while lower values of S predict bone tissue formation as follows:

3 $$\begin{array}{ll}{\rm{Mature}}\,{\rm{trabecular}}\,{\rm{bone}} & 0.0000 < {\rm{S}} < 0.2667\\ {\rm{Immature}}\,{\rm{woven}}\,{\rm{bone}} & 0.2667 < {\rm{S}} < 1.0000\\ {\rm{Cartilaginous}}\,{\rm{tissue}} & 1.0000 < {\rm{S}} < 3.0000\\ {\rm{Fibrous}}\,{\rm{tissue}} & 3.0000 < {\rm{S}}\end{array}$$

The corresponding material properties of these tissue types are given in Table 2. During healing, the local cell concentration depends on diffusion. An effective value for a given material property M_e_ in the healing region is found by using the rule of mixtures as follows:

4 $${{\rm{M}}}_{{\rm{e}}}({\rm{t}})=\frac{{{\rm{n}}}_{{\rm{s}}{\rm{c}}}}{{{\rm{n}}}_{{\rm{s}}{\rm{c}}}^{(max)}}{\rm{M}}({\rm{t}})+(1-\frac{{{\rm{n}}}_{{\rm{s}}{\rm{c}}}}{{{\rm{n}}}_{{\rm{s}}{\rm{c}}}^{(max)}}){{\rm{M}}}_{{\rm{g}}}$$

where M_e_ is one of the six material properties listed in Table 2, M(t) is the local poroelastic property that evolves as described by Eq. (3) and M_g_ is the property for the granulation tissue.

**Table 2:** Poroelastic properties of tissues from Lacroix and Prendergast^23^.

	Elastic Modulus (GPa)	Poisson’s Ratio	Porosity	K_s_ (GPa)	K_f_ (GPa)	Permeability (mm^4^/(N.s))
Granulation Tissue	0.002	0.17	0.8	2.3	2.3	0.01
Fibrous Tissue	0.002	0.17	0.8	2.3	2.3	0.01
Cartilage	0.01	0.17	0.8	3.4	2.3	0.005
Immature Woven Bone	1	0.30	0.8	13.92	2.3	0.1
Mature Trabecular Bone	3.5	0.30	0.8	13.92	2.3	0.37
Cortical Bone	14.5	0.30	0.04	13.92	2.3	10^−5^

The evolution of material properties during healing depends on the concentration of available cells, the mechanical response of the poroelastic tissue and the stimulus described above. The time dependent nature of this coupled problem is solely due to the diffusion equation in this model. Equations (1–4) are solved through numerical iterations where each iteration is considered to be one day long. Due to the rapid changes in material properties during this pseudo-transient solution, numerical damping is introduced by using a moving average of the predicted quantities^23^. Recently we used the following relaxation approach as an alternative to the moving average in order to dampen the abrupt changes in material properties:

5 $${{\rm{M}}}_{{\rm{e}}}^{({\rm{i}}+1)}={{\rm{M}}}_{{\rm{e}}}^{({\rm{i}})}+\alpha ({{\rm{M}}}_{{\rm{e}}}({\rm{t}})-{{\rm{M}}}_{{\rm{e}}}^{({\rm{i}})}),\,\alpha =0.2$$

where i represents the solution iteration level (i.e., day). Effects of initial material properties of the granulation tissue, geometrical properties, and MSC diffusion constant, on the healing pathway have been studied by Ghiasi *et al*.^48^. Another parametric study performed by the authors^30^ shows that the D and α values used in the present work are effective in damping out the spurious fluctuations. In this work the material properties were updated in the healing gap for 30 iterations, and final values were used as the initial conditions at the start of bone remodeling phase.

During fracture healing, the implant was subjected to oscillatory displacement according to the haversine function:

6 $${\rm{z}}({\rm{t}})=\frac{1}{2}{{\rm{z}}}_{{\rm{\max }}}(1-\,\cos \,2{\rm{\pi }}{\rm{\nu }}t)$$

where z_max_ is the range of the micromotion, with an amplitude of ± ${{\rm{z}}}_{{\rm{\max }}}/2$. The oscillation frequency ${\rm{\nu }}$ was kept constant at 1 Hz. z_max_ values of 5, 10 and 20 μm were used in this work. Numerical experiments showed that simulation duration of 4 seconds was sufficient to find the steady state conditions. Average of fluid velocity and shear strain during transient solution was used to calculate healing stimulus in Eq. (2). The effects of loading rate, abrupt changes in loading conditions and calculation of the accumulated healing stimulus on the prediction of fracture healing pathway will be discussed in separate articles.

### Bone remodeling

Bone needs a certain level of mechanical stimulation to maintain an apparent density distribution that can withstand the daily loading cycles^49^. The apparent density of bone does not change if the homeostatic stimulus level can be maintained. Loading levels that cause stimulus higher than the homeostatic level cause the apparent bone density to increase. At very high loading levels the bone can fracture. On the other hand, loading levels that result in stimulus lower than the homeostatic levels can cause the bone to resorb. These effects are carried out in two ways. Bone adapts both its shape (surface remodeling) and its internal material properties (internal remodeling) through cellular activities of osteoclasts removing dead bone cells and osteoblasts depositing new cells^50,51^. In the present study internal changes in bone material properties in response to the mechanical environment is investigated. Although it has been shown that bone remodeling depends on the fluid velocity as well as the mechanical signals in the solid phase^52^, only the latter one was used in this work and bone and other tissue types were modeled as isotropic elastic materials. Using a poroelastic model during bone remodeling, where updating porosity affects the fluid flow in the trabecular bone and its mechanical behavior in general, should be considered in the future.

In nature and in this work, the end state of the healing phase serves as the initial condition of the remodeling phase. Assuming that soft tissue cannot remodel, only the healed regions with elastic modulus of 100 MPa and higher were allowed to remodel, or in other words, experience adaptation of the apparent density. This phase of adaptation was simulated by using Carter *et al*.’s model^49^, which calculates the rate of change of the apparent bone density as follows:

7 $$\dot{{\rm{\rho }}}=\dot{{\rm{r}}}\,{{\rm{S}}}_{{\rm{v}}}\,{{\rm{\rho }}}^{(\max )}$$

where ${\rm{\rho }}$ is the apparent bone density, ${{\rm{\rho }}}^{({\rm{\max }})}=1.92\,{\rm{gr}}/{{\rm{cm}}}^{3}$ is the density of fully mineralized bone tissue with zero porosity and S_v_ is bone specific surface (BS/TV) calculated from:

8 $${{\rm{S}}}_{{\rm{v}}}=0.6255\,{\rho }^{6}-3.703\,{\rho }^{5}+7.0228\,{\rho }^{4}-4.8345\,{\rho }^{3}-1.928\,{\rho }^{2}+6.745\,\rho $$

In Eq. (7), $\dot{{\rm{r}}}$ is the linear rate of bone apposition or resorption^49,53^ that is represented as follows:

9 $$\dot{{\rm{r}}}=\left\{\begin{array}{cc}{{\rm{c}}}_{{\rm{r}}}\,(\psi -{\psi }_{{\rm{A}}{\rm{S}}})+{{\rm{c}}}_{{\rm{r}}}\,{\rm{w}} & \psi -{\psi }_{{\rm{A}}{\rm{S}}} < -{\rm{w}}\\ 0 & \,-{\rm{w}} < \psi -{\psi }_{{\rm{A}}{\rm{S}}} < {\rm{w}}\\ {{\rm{c}}}_{{\rm{f}}}(\psi -{\psi }_{{\rm{A}}{\rm{S}}})-{{\rm{c}}}_{{\rm{f}}}\,{\rm{w}} & {\rm{w}} < \psi -{\psi }_{{\rm{A}}{\rm{S}}}\end{array}\right.$$

where ${{\rm{c}}}_{{\rm{r}}}=2\times {10}^{-5}$ and ${{\rm{c}}}_{{\rm{f}}}=2\times {10}^{-4}$ are the rates of bone resorption and bone apposition with respect to daily stimulus ${\rm{\psi }}$. ${{\rm{\psi }}}_{{\rm{AS}}}=15\,{\rm{MPa}}/{\rm{day}}$ is defined as attractor (or homeostatic) stress stimulus for 112 number of daily load cycles^49^. ${\rm{w}}=0.25{{\rm{\psi }}}_{{\rm{AS}}}$ is half width of the dead zone in which bone maintains its density ($\dot{{\rm{r}}}=0)$. The daily stress stimulus is defined by using a tissue-level measure as follows:

10 $$\psi ={({\rm{N}}{{\sigma }_{{\rm{T}}}}^{{\rm{m}}})}^{1/{\rm{m}}}$$

where N is number of cycles of loading and in this work a value of m = 4 was used based on experimental data for cortical and cancellous bone^54^. The tissue level stress ${{\rm{\sigma }}}_{{\rm{T}}}$ is related to the continuum level stress ${{\rm{\sigma }}}_{{\rm{c}}}$ as follows:

11 $${\sigma }_{{\rm{T}}}={\left(\frac{{\rho }^{(max)}}{\rho }\right)}^{2}{\sigma }_{{\rm{c}}}$$

The continuum level stress can be represented by using the elastic modulus E and strain energy density u of the material as follows:

12 $${{\rm{\sigma }}}_{{\rm{c}}}=\sqrt{2\,{\rm{Eu}}}$$

Bone elastic modulus is related to its apparent density^49^ by the following empirical relationship:

13 $${\rm{E}}=\{\begin{array}{c}2042.82\,{{\rm{\rho }}}^{2.5},\,{\rm{\rho }} < 1.2\\ 1798.06\,{{\rm{\rho }}}^{3.2},\,{\rm{\rho }} > 1.2\end{array}$$

Equations (7–13) are solved numerically. In particular, Eq. (7) is discretized by using the forward time integration scheme. Each time step represents 30 days of bone loading and Eq. (10) is adjusted accordingly. The daily remodeling stimulus was determined by using N = 112 for a load value of 100 N on the tooth as the typical daily mastication regime.
